# Percepção da prematuridade por familiares na unidade neonatal: estudo Transcultural**

**DOI:** 10.15649/cuidarte.1043

**Published:** 2022-08-06

**Authors:** Ana Celi Silva Torres Nascimento, Aisiane Cedraz Morais, Sinara de Lima Souza, Maria Carolina Ortiz Whitaker

**Affiliations:** 1 . Universidade Estadual de Feira de Santana, Feira de Santana, Bahia, Brasil. E-mail: celitorres19@hotmail.com Universidade Estadual de Feira de Santana Universidade Estadual de Feira de Santana Feira de Santana Bahia Brazil celitorres19@hotmail.com; 2 . Universidade Estadual de Feira de Santana, Feira de Santana, Bahia, Brasil. E-mail: aisicedraz@hotmail.com Universidade Estadual de Feira de Santana Universidade Estadual de Feira de Santana Feira de Santana Bahia Brazil aisicedraz@hotmail.com; 3 . Universidade Estadual de Feira de Santana, Feira de Santana, Bahia, Brasil. E-mail: sinarals@uefs.br Universidade Estadual de Feira de Santana Universidade Estadual de Feira de Santana Feira de Santana Bahia Brazil sinarals@uefs.br; 4 . Universidade Federal da Bahia, Salvador, Bahia, Brasil. E-mail: maria.ortiz@ufba.br Universidade Federal da Bahia Universidade Federal da Bahia Salvador Bahia Brazil maria.ortiz@ufba.br

**Keywords:** Família, Recém-Nascido Prematuro, Unidades de Terapia Intensiva Neonatal, Enfermagem Transcultural, Family, Infant, Premature, Intensive Care Units, Neonatal, Transcultural Nursing, Familia, Recién Nacido Prematuro, Unidades de Cuidados Intensivos Neonatales, Enfermería Transcultural

## Abstract

**Introdução:**

Conheceraprematuridadepelapercepçãodasfamílias de recém-nascidos internados na Unidade de Terapia Intensiva Neonatal sob a perspectiva Transcultural.

**Materiais e Métodos:**

Pesquisa qualitativo, descritiva e exploratória. Foram realizadas Observação participante e entrevistas semiestruturadas com 16 familiares de recém-nascidos internados na unidade neonatal de uma maternidade pública do interior baiano. Utilizou-se o *software Interface de R pour analyses Multidimensionnelles de Textes et de Questionnaires*® e análise de conteúdo.

**Resultados:**

Emergiram quatro categorias: O prematuro em suas particularidades na percepção da família; Sentimentos familiares ocasionados pela prematuridade; A espiritualidade como estratégia para significar a prematuridade; A prematuridade por meio dos cuidados profissionais.

**Discussões:**

Compreender as experiências das famílias no ambiente neonatal pode contribuir com os profissionais de saúde e instituições na reorientação às suas práticas, visando uma assistência voltada para integralidade, identificando as singularidades de cada família e o reconhecimento da diversidade cultural.

**Conclusão:**

Entender a família, em suas necessidades e cultura, colabora com uma assistência mais humana e eficaz, onde a família fará parte do planejamento e da tomada de decisão para cuidar do prematuro amplamente, respeitando o biológico, como também, fatores sociais e psicológicos, contemplando integralmente, o prematuro e sua família.

## Abstract (en)

**Introduction:**

To understand premature birth through perceptions of families with premature babies admitted to neonatal intensive care unit from a cross-cultural perspective.

**Materials and methods:**

An exploratory-descriptive qualitative study was conducted through participant observation and semi-structured interviews with 16 relatives of premature babies admitted to the neonatal intensive care unit of a maternity hospital in the state of Bahia. R Software was used for multidimensional analysis of texts and questionnaires and content analysis.

**Results:**

Four categories were established: premature babies with their particularities in family’s perceptions; family’s feelings caused by premature birth; spirituality as a strategy to give meaning to premature birth; and premature birth from professional healthcare.

**Discussion:**

Understanding the experiences of families in the neonatal environment can contribute to healthcare professionals and institutions reorienting their practices to target integrality of care by identifying the particularities of each family and recognizing their cultural diversity.

**Conclusions:**

Understanding the needs and culture of families contributes to the provision of more humane and effective care in which the family plays a part in planning and decision making on integral care of their premature baby, not only highlighting biological factors but also social and psychological factors that comprehensively involve the premature baby and their family.

## Resumen

**Introducción:**

Entender la prematuridad a través de la percepción de las familias de los recién nacidos ingresados en una Unidad de Cuidados Intensivos desde una perspectiva transcultural.

**Materiales y métodos:**

Investigación cualitativa, descriptiva y exploratoria. A través de una observación participante, se llevaron a cabo entrevistas semiestructuradas con 16 familiares de recién nacidos prematuros hospitalizados en la unidad neonatal de un hospital materno en el estado de Bahía. Se hizo uso del software Interfaz de R para el análisis multidimensional de textos y cuestionarios, y análisis de contenido.

**Resultados:**

Se establecieron cuatro categorías: el recién nacido prematuro con sus particularidades en la percepción de la familia; sentimientos en la familia generados por la prematuridad; la espiritualidad como estrategia para darle significado a la prematuridad; la prematuridad atendida a través del cuidado profesional.

**Discusión:**

La comprensión de las experiencias de las familias en el ambiente neonatal puede contribuir a los profesionales e instituciones de salud a reorientar sus prácticas, a fin de apuntar a una atención centrada en la integralidad al identificar las particularidades de cada familia y reconocer su diversidad cultural.

**Conclusiones:**

Entender las necesidades y la cultura de las familias contribuye a prestar una atención más humana y eficaz en la que la familia hace parte de la planificación y toma de decisiones en el cuidado integral del recién nacido prematuro al no resaltar solamente los aspectos biológicos, sino también los factores sociales y psicológicos que incluyen al recién nacido prematuro y su familia de una forma integral.

## Introdução

O prematuro é definido como todo recém-nascido (RN) proveniente de nascimentos após a 20ª e anterior à 37ª semana de gestação. A prematuridade ou as condições a ela associadas, tem sido considerada uma questão de saúde pública pelo número de crianças prematuras que nascem anualmente no mundo e pelos altos índices de morbidade e mortalidade no período neonatal^1^.

Nos últimos anos, houve um aumento na prevalência mundial, onde 15 milhões de RN nasceram prematuramente, representando uma incidência mundial de 11,1% dos nascimentos, sendo o Brasil foi considerado o décimo (10º) país com maior número de casos estimados de prematuridade^1^. Atualmente, segundo os dados do Nascer no Brasil, que pesquisou sobre parto e nascimento, ocorreram 11,5% partos de bebês prematuros, porcentagem que corresponde a quase duas vezes superior ao observado em países europeus^2^.

Nascer prematuramente exige do RN adaptação ao meio extrauterino devido as suas características particulares, envolvendo imaturidade orgânica e fisiológica, sendo necessária uma assistência habitualmente realizada em Unidade de Terapia Intensiva Neonatal (UTIN) que demandam cuidados especializados, recursos tecnológicos e humanos adequados. Onde oportuniza o tratamento das doenças neonatais e auxilia na redução da mortalidade neonatal, sendo fonte de esperança na recuperação do prematuro^3^.

A necessidade de tratamento intensivo ocasionada pelo nascimento precoce gera no bebê e sua família o enfrentamento da hospitalização e a separação precoce e prolongada, na qual pode interferir na formação de laços afetivos e esta separação pode provocar um distanciamento entre pais e bebê, acarretando consequências deletérias para o desenvolvimento global do RN^4^. Além de poder acionar a falta de confiança na capacidade de cuidar do filho, deixando de viver o papel parental no ambiente neonatal.

Ter um filho prematuroé uma experiência desafiadora que altera a dinâmica familiar, somado com a hospitalização, pode gerar uma interrupção na regularidade da vida da família, constituindo em um momento repleto de dificuldades, frustrações e medos, exigindo uma série de tomadas de decisão que, normalmente, a família não tem amadurecimento suficiente para enfrentar esta problemática. Esta ruptura da unidade familiar leva ao desequilíbrio na sua capacidade de funcionamento, ocasionando conflitos e afastamento dos seus membros^5^.

A participação dos profissionais de saúde no apoio ao fortalecimento de uma ligação forte e segura com a família, minimizando ao máximo a separação do RN com seus pais, proporcionando um ambiente receptivo e acolhedor para ambos é primordial prestar uma assistência a todos os familiares e não somente ao RN; pois, o vínculo daqueles que cuidam da criança, geralmente mãe e pai, é crucial para a sobrevivência e desenvolvimento do prematuro^6^.

Para o cuidado ao prematuro e sua família, os profissionais de saúde precisam compreender as necessidades apresentadas pelos familiares para que possam planejar e promover assistência eficaz, observando a singularidade de cada caso, envolvendo aspectos biológicos, sociais e espirituais, pois cada familiar tende a reagir influenciado por sua cultura e vivências, proporcionando uma assistência embasada no reconhecimento das diversas culturas existentes^7^^,^^8^.

A partir do exposto, o estudo teve como objetivo conhecer a prematuridade pela percepção das famílias de recémnascidos internados na Unidade de Terapia Intensiva Neonatal sob a perspectiva Transcultural.

## Materiais e Método

Estudo qualitativo, descritivo e exploratório, sendo fundamentado na Teoria Transcultural de Leininger. Onde atendeu aos passos recomendados pelos Critérios Consolidados para Relatar uma Pesquisa Qualitativa (COREQ)^9^.

A Teoria Transcultural focaliza o estudo comparativo e a análise de variadas culturas, no que é relativo ao cuidado de enfermagem de qualidade e humanístico. Sendo dividida em quatro níveis, porém o foco desta investigação é a segunda etapa, que envolve os indivíduos, famílias e culturas no contexto do sistema de saúde, no qual defende a importância e a influência da dimensão cultural na interconexão do cuidado aplicado na enfermagem, que é essencial para uma assistência ampla, eficiente e resolutiva.

Neste estudo, foi considerada família como uma unidade dinâmica, unida por laços de consanguinidade e/ou afetividade, considerando pessoas próximas como membros da família (avós, amigos e vizinhos)^10^. Foi realizado entrevista com 16 familiares de prematuros internados na UTIN de uma maternidade do interior baiano, abordados no ambiente neonatal e encaminhados para uma sala reservada da coordenação de enfermagem.

Foram adotados critérios de inclusão: famílias de RN pré-termos; internados na UTIN há pelo menos sete dias, considerando ser um tempo ideal de convivência que possibilite responder ao questionamento do objeto de estudo. A faixa etária dos participantes foram pais e mães adolescentes com a idade igual ou superior a 15 anos; e membros da família ampliada acima dos 18 anos. Foram excluídos: famílias de prematuros que tivesse associado alguma má-formação congênita.

Para a coleta de dados foram utilizadas as técnicas de observação participante e a entrevista semiestruturada, com duração média de 18 minutos, entre maio e junho de 2019. Ressalta-se que as entrevistas realizadas foram gravadas com a utilização de aparelho eletrônico/digital mediante a autorização dos participantes que concordaram em participar da pesquisa e após a assinatura dos devidos Termos de Consentimento.

Para manter o anonimato, os fragmentos de cada familiar entrevistado apareceram codificados pela sequência da letra E, seguida de um algarismo numérico para representar a ordem de participação.

Posteriormente, os dados foram avaliados pelo método de Análise de Bardin, levando em consideração a análise de conteúdo, onde a técnica consta de três etapas fundamentais: a préanálise, a exploração do material e o tratamento dos resultados^11^.

Para complementar a análise de conteúdo de Bardin, foi utilizado o *software* denominado *Interface de R pour analyses Multidimensionnelles de Textes et de Questionnaires*® (IRAMUTEQ®), na versão 0.7, alpha 2, sendo um programa que permite análises estatísticas sobre os corpus textuais e sobre tabelas indivíduos/palavras^12^.

Das análises de dados textuais oferecidas por esse *software*, optou-se por:“análise de similitude”. Este tipo de análise permite identificar as coocorrências entre as palavras e o seu resultado traz indicações da conexidade entre as palavras, auxiliando na identificação da estrutura do conteúdo de um corpus textual^13^.

Todas as etapas desta pesquisa foram respaldadas pelas normas e diretrizes estabelecidas pela Resolução nº 466, de 12 de dezembro de 2012, do Conselho Nacional de Saúde, órgão nacional regulador que atende os princípios éticos estabelecidos em pesquisa envolvendo seres humanos. A pesquisa foi submetida à apreciação do Comitê de Ética e Pesquisa da Universidade Estadual de Feira de Santana, sob o parecer favorável nº 3.218.290/2019 e o CAAE: 06704919.6.0000.0053.

## Resultados

Participaram do estudo 16 familiares: 14 mães, um pai e um vizinho/amigo da família. Quanto à idade, variou de 17 a 46 anos. Sobre o estado civil, oito (50%) eram solteiros, três (19%) declararam-se casados, quatro (25%) em união consensual e uma (6%) mulher divorciada. Quanto à religião, oito (50%) declararam serem católicos, quatro (25%) evangélicos e quatro (25%) sem religião, porém afirmaram acreditar em Deus.

Ao utilizar o programa Iramutec, a Análise de Similitude permitiu gerar o gráfico abaixo, onde ilustra as relações entre as principais palavras ou formas que compõem os seguimentos de texto das entrevistas.

**Figura 1:**
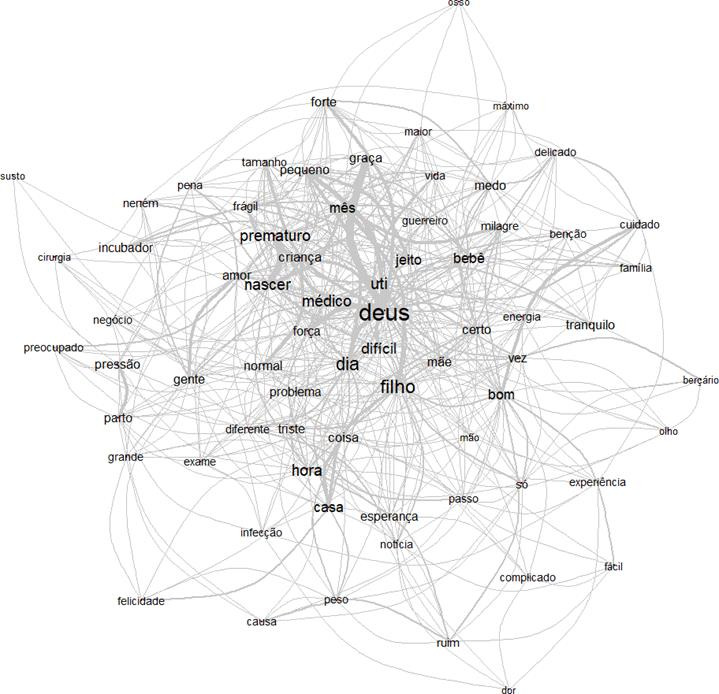
Análise de Similitude sobre a prematuridade na visão dos familiares.

*Fonte: Dados da investigação analisado pelo software IRAMUTEQ.*

A partir dessa análise baseada na teoria dos grafos é possível identificar as ocorrências entre as palavras e as indicações de conexidade entre estas, auxiliando na identificação da estrutura do conteúdo de um corpus textual^12^. Observa-se as palavras de mais destaque nos discursos:“Deus”, “filho”e“prematuro”; e delas se ramificam outras que apresentam expressões significativas, como: “UTI”, “médico”, “difícil”, “força”, “frágil” e “amor”. Na periferia apareceram as palavras: “pequeno”, “milagre”, “energia”, “triste”, “diferente” e “pena”; e nos extremos das ramificações contemplam as relações entre: “forte” e “delicado”; “cuidado” e “família”; “dor” e “felicidade”.

Neste sentido, pode-se inferir quede uma forma geralos discursos dos participantes, além de apresentarem referências que são inerentes ao processo da prematuridade para a família, como a relevância da presença de Deus para vivenciar as peculiaridades do filho prematuro, considerando a espiritualidade como suporte para vivenciar esta fase inesperada e complicada para a família; além da necessidade da hospitalização na UTIN e da colaboração dos profissionais de saúde, onde é relacionando a figura do “médico” como possibilidade de sobrevivência ao prematuro.

Dos fatores presentes no segundo nível do Modelo Sunrise de Leininger, foi possível identificar e descrever com mais evidência os fatores religiosos, culturais e educativos que influenciam os padrões e expressões de cuidado e saúde relacionados à prematuridade^14^. Esses fatores se entrelaçam e afetam diretamente no cuidado de enfermagem, possibilitando uma assistência ampla, eficiente e resolutiva.

Os resultados foram decodificados, gerando quatro categorias: O prematuro em suas particularidades; Sentimentos familiares ocasionados pela prematuridade; A espiritualidade como estratégia para significar a prematuridade; e A prematuridade por meio dos cuidados profissionais.

### O prematuro em suas particularidades

Foi possível identificar que as famílias identificam o prematuro como um ser muito pequeno, frágil, delicado; mas, ao mesmo tempo, “fortes” e “espertos”. Desta forma, encontram forças para vivenciar e superar a hospitalização:

*“*É f*rágil, é guerreiro também, né! (...) Que ele é muito pequeno, fico toda sem jeito de pegar (...). Tem uma força retada”. [E2]*

*“*É está sendo forte até demais, tá sendo um guerreiro*”. [E6]*

A partir do reconhecimento de que o bebê prematuro tem características específicas, estes familiares também pontuam a necessidade de cuidados diferenciados, que possam atender as particularidades da prematuridade; ainda ressaltando a sua importância na recuperação, através da sua presença e participação no cuidado ao filho no ambiente neonatal:

*“Que ele depende de mim o máximo dos máximos, dos máximos. Eu sou o ponto chave pra recuperação dele”. [E11]*

*“Um bebê muito delicado que você tem que ter o máximo de cuidado possível (...) Você pegar pra dar a mama, dar o banho, trocar a fralda (...). Você fica querendo ter ele pra você o tempo todo”. [E15]*

Além disso, emergiu que a hospitalização do prematuro imprime modificações positivas no ambiente familiar, reforçando os vínculos e as relações, contrapondo com achados da literatura, que valorizam o lado negativo da hospitalização:

*“Ele mudou a minha vida (...). Ele me mudou totalmente, tanto com a minha família, quanto comigo mesma”. [E5]*

### Sentimentos familiares ocasionados pela prematuridade

Uma peculiaridade verificada nesta categoria foi que a prematuridade exige paciência da família para enfrentar e suportar de forma menos traumática o internamento do prematuro na UTIN; uma vez que esta condição se distancia do que esperavam: um nascimento a termo e com o bebê ao seu lado em Alojamento Conjunto:

*“Significa ter muita paciência (...). Com tudo assim, com a situação, com o fato dele tá ali e você não poder ter o seu filho com você o tempo todo, do seu lado”. [E15]*

*“A prematuridade é complicada, né! Porque é uma fase que deveria ter tido ele formado por completo. Aí você vê ele daquele jeitinho, né! Bom sinal não é”. [E7]*

Após o nascimento do filho, a prematuridade representa um momento complexo na vida das famílias; pois, esta experiência provoca um misto de sentimentos, como insegurança, sofrimento, impotência e compaixão; além de culpa pela criança está vivenciando a hospitalização:

*“Nossa é um negócio muito, muito difícil de lhe dar, muito difícil mesmo”. [E14]*

*“*É primeiro a gente sente pena, né. Pelo tamanho, tamanhozinho, o sofrimento. A gente, eu fico com pena. Não vou mentir*”. [E6]*

*“Eu sinto culpa também, mas eu não sabia (...). Eu sofro com isso também. Porque era para ele nascer forte, com nove meses dele”. [E6]*

Foi enfatizado também que a prematuridade provoca muito medo na família devido à instabilidade da condição do RN, a qual apresenta situações extremas que vão desde o risco iminente de morte à esperança de melhora e sobrevivência do filho:

*“Você fica assim, aquele medo de qualquer coisa ele adoecer, porque não tem os órgãos totalmente formado como os outros bebês (...). E você fica com medo de pegar, ser prematuro”. [E15]*

Outro aspecto desvelado é que a prematuridade pode ser uma experiência inicialmente que assusta e emociona os pais, associada à ruptura da conexão; entretanto, as falas reportam há um vínculo fortalecido pelo sentimento de amor entre pais e filhos:

*“*É uma novidade, uma experiência, não é! Porque eu pensava que eu ia ter ela no tempo certo, né! Só que tive com seis meses. Aí, foi um susto, um susto mesmo*”. [E10]*

*“Representa um amor né, de mãe. Um carinho enorme que a gente passa a ter (...). Como ele está na incubadora, assim, é um amor imenso”. [E16]*

### A espiritualidade como estratégia para significar a prematuridade

Ao observar o ambiente neonatal e os discursos, pode-se evidenciar o quanto a fé demonstrada pelas famílias é vital para significar a prematuridade e vivenciar a hospitalização. De acordo com Leininger, crenças e valores culturais influenciam os padrões e expressões de cuidado e saúde^14^; interferindo como esses familiares entendem a prematuridade e processo do internamento.

*“*É difícil, mas a gente busca forças em Deus, com esperança que vai sair logo

*(...). Pedindo forças a Deus pra que dê forças a ela pra suportar”. [E9]*

*“Porque a gente queria está com ele no colo, cheirar, beijar, e é assim, né. Só pedir forças a Deus (...). Tá na mão de Deus”. [E16]*

Muitas famílias vinculam a recuperação e a possibilidade de alta hospitalar da criança como primeiramente a um milagre divino. As falas seguintes demonstram esses aspectos culturais:

*“Um milagre porque nos tempo antigos minha avó falava que um prematuro não sobrevivia, ele nascia e morria. Ele só dava o primeiro choro e morria”. [E12]*

*“*É, uma criança nascer prematura e ficar bem, é só Deus mesmo. Que nem todos conseguem ainda sobreviver, mas graças a Deus todos ali tá reagindo*”. [E5]*

### A prematuridade concebida pelos cuidados profissionais

Na análise de similitude, a palavra médico emerge quase centralizada, sugerindo a imponência dos cuidados profissionais para a significação que familiares trazem à prematuridade, associada ao suporte tecnológico que a UTIN dispõe para esses bebês:

*“Através da medicina que tá dando suporte para poder esses bebês conseguirem sobreviver através dos medicamento e acompanhamentos”. [E12]*

Os cuidados e orientações dispensados à família pelos profissionais de saúde são referenciados como essenciais à condição inerente a prematuridade, trazendo à tona elementos pontuados por Leininger ao analisar os modos de cuidar, como fatores tecnológicos, inter-relacionamentos, suporte social e educacional^8^, suscitando que o cuidado congruente é possível na medida em que as falas abaixo elucidam que o atendimento das necessidades dos bebes e das famílias foram potencialmente atendidas:

*“No começo fiquei bem depressiva, mas tive muita (suporte) da equipe médica que melhorou bastante aqui dentro. (...) Os psicólogos viam conversar comigo (...), as médicas, até os estagiários vinham, sempre conversavam comigo. Me dava conselho, me ajudou bastante”. [E2]*

*“Depois as enfermeiras, os médicos foram me acalmando, falando que ele tá bem, sempre me dão o boletim (médico) todos os dias. Aí foi me aliviando mais, aí agora estou tranquila”. [E5]*

Contraditoriamente, uma mãe refere uma atitude profissional que a deixou temerário diante das incertezas da prematuridade:

*“Tem aquele médico que passa segurança. Tem uns que fica: “É tem chance, né!”. Aí você fica toda sem, não sente segurança”. [E3]*

Neste sentido, há certa irregularidade na forma de como acolher e atender as necessidades de cada família pelos profissionais de saúde; gerando insegurança, insatisfação e o comprometimento da autonomia familiar no ambiente neonatal, reforçando a necessidade de apropriação do modelo de assistência Cuidado Centrado na Família (CCF) que contempla o RN e sua família como uma unidade de cuidado.

## Discussão

O prematuro apresenta características peculiares e queassociado ao fato dos pais não esperarem o nascimento de uma criança fragilizadaacaba se tornando complexo para a família, que se depara com um RN pequeno e imaturo, diferente daquele imaginado durante a gestação^15^^,^^16^. Porém, neste estudo, verificou-se que a família além de reconhecer o RN como “miúdo” e “frágil”, também conseguiu enxergá-lo como “forte” pela potencialidade de enfrentar a hospitalização. Do ponto de vista transcultural, o bebê idealizado distancia-se da imagem do prematuro, o que impõe mais dificuldades para esses familiares significarem a prematuridade com tranquilidade.

Evidenciou-se que ter um RN precoce e que necessita permanecer hospitalizado é uma experiência difícil e marcante para as famílias, devido à interrupção de sonho de ter um filho saudável, a termo e que permaneça junto à família logo após o nascimento^17^. Assim, é uma vivência dolorosa e cheia de expectativas, que exige muita paciência por parte dos familiares; pois, o internamento pode provocar desestruturação familiar, alterando a sua organização, impondo-lhes a necessidade de reorganizar o seu funcionamento para o enfrentamento dessa nova situação^18^.

As mães e os pais e demais familiares de recém-nascidos de risco devem ser assistidas pelos profissionais de saúde munidos de elementos que viabilizam a prática de um cuidado singular, centrado nas crenças, valores e estilos de vida de cada mulher e sua família como é defendido pela Teoria Transcultural de Leininger^18^.

O ambiente hospitalar e a aparência física do prematuro ocasionam nas famílias um misto de sentimentos ambivalentes evidenciado por dúvidas, decepção, culpa, insegurança, sofrimento e medo pelo filho hospitalizado; porém, ao mesmo tempo demonstram contentamento com o nascimento do filho, situação em que potencializa o amor, emoções positivas e confiança em sua recuperação^17^^,^^19^.

Porém, existe a prevalência neste estudo de sentimentos negativos, como a inexperiência e incerteza de prestar cuidados maternos à fragilidade do prematuro, que dificultam a interação e o cuidar do filho da forma desejada e sonhada^20^^,^^21^. Para a família, estar na UTIN com um prematuro, principalmente a mãe, faz com que ela sinta perda de sua função materna, tento dificuldade de se reconhecer no ambiente neonatal, uma vez que existe uma equipe de saúde quena maioria das vezesse apropria dos cuidados que deveriam ser realizados pela família, deixando-a muitas vezes como expectadora no ambiente neonatal^22^.

Outros fatores que desencadeiam medo e angústia nas famílias são por estar numa UTIN, local este que significa ameaça iminente de morte, onde desvela-se a incerteza da vida do RN. Além da instabilidade do quadro clínico e pela experiência desconhecida de vivenciar a hospitalização, onde o período inicial é mais difícil e doloroso^23^^,^^24^, relatado por alguns entrevistados desta pesquisa.

O sofrimento pela hospitalização acentua-se em nossa cultura do “não sofrimento”, na qual a busca pela felicidade se traduziria mais pelo evitar do sofrimento do que pela vivência de fortes prazeres; pois, vivemos em uma cultura da analgesia, em que não basta minimizar o sofrimento, mas este deve ser abolido. Por isto, devemos sempre resgatar a teoria de Leininger^14^, pois os modos de cuidar se ressignificam nesta cultura da “analgesia”^25^^-^^26^.

Com passar do tempo e com a reorganização da rotina familiar, as famílias aprimoram os seus entendimentos e passam acreditar na possibilidade da alta hospitalar e a enxergar a UTIN como um local adequado para cuidar do seu filho, permitindo que os sentimentos como segurança e tranquilidade se sobressaiam aos episódios de medo e ansiedade^20^.

Uma maneira da família (re) significar a prematuridade e vivenciar esta situação de forma mais amena, apontada nesta pesquisa, é através do apoio emocional e educacional dos profissionais de saúde que ajudam a família a fortalecer o vínculo de forma gradual com o RN^16^. Neste sentido, os profissionais estabelecem escuta e comunicação efetiva com as famílias, tornando esta experiência menos sofrida, estabelecendo uma relação de empatia entre RN/família/equipe de saúde.

Verifica-se que a família vive uma dualidade de sentimentos, onde se apegam principalmente a aspectos positivos para experienciar a hospitalização do prematuro, como também, sem esquecer os aspectos dolorosos. Os resultados apontam que a valorização dos cuidados profissionais aos bebês funcionou para confortar a família, deixando-os satisfeitos ao perceber a dedicação da equipe dispensada ao filho e também por acolhê-los, minimizando a ansiedade e o medo, sendo esses cuidados essenciais à condição inerente da prematuridade.

As famílias também buscam forças na dimensão espiritual, através da fé em Deus para conseguir experienciar as particularidades da prematuridade. Onde vivem na linha tênue entre a vida e a morte, entre o cuidar do filho e abrir mão deste cuidado permitindo que um profissional de saúde execute em seu lugar, extrapolando o cotidiano e a dimensão biológica, na espera do sucesso na terapia e da alta hospitalar^27^^-^^28^.

## Conclusão

Pelos discursos dos familiares, pode-se compreender como estes significam a prematuridade e processos inerentes a esta, como os aspectos biológicos que dizem respeito às características do prematuro, a exigência de cuidados especializados, a centralidade da espiritualidade, colocando Deus como suporte para entender e vivenciar a hospitalização; além de retratar a prematuridade por meio da hospitalização na UTIN e dos cuidados do profissionais de saúde, onde relaciona-se a figura do “médico” como possibilidade de sobrevivência desta criança.

Compreender as experiências das famílias no ambiente neonatal pode contribuir com os profissionais de saúde e instituições na reorientação às suas práticas, visando uma assistência pautada em tecnologias leves, através do acolhimento, responsabilização e pelo respeito à vida, através da perceptiva da integralidade, identificando as singularidades de cada família e o reconhecimento da diversidade cultural referenciada por Leininger.

Entende-se como limite dessa pesquisa a eleição de uma única instituição com familiares de prematuros, retratando assim uma realidade local. Sendo necessária a investigação em outros cenários que possam apontar outras práticas ou outros contextos transculturais.
